# Applying behaviour change science to change motivational interaction in physical activity and sports professionals: stepwise development and acceptability pre-test of the MotiStyleSport intervention

**DOI:** 10.1080/21642850.2026.2716945

**Published:** 2026-10-09

**Authors:** Minttu Palsola, Elina Renko, Melina Puolamäki, Pilvikki Absetz, Leen Haerens, Taru Lintunen, Sebastian Potthoff, Kaisa Saurio, Nelli Hankonen

**Affiliations:** a Faculty of Social Sciences, Tampere University, Tampere, Finland; b Department of Movement and Sports Sciences, Faculty of Medicine and Health Science, Ghent University, Ghent, Belgium; c Faculty of Sport and Health Sciences, University of Jyväskylä, Jyväskylä, Finland; d School of Communities and Education, Northumbria University, Newcastle upon Tyne, UK

**Keywords:** Self-determination theory, habit theory, intervention development, behaviour change, physical activity

## Abstract

**Background:**

Physical activity (PA) counselling and sports coaching are more effective when professionals use self-determination theory-based need-supportive interaction. Existing interventions generally involve information provision and practical exercise, and show modest, short-term effects. Behaviour change science has rarely been incorporated, despite its potential to support sustainable change.

**Aims:**

This study systematically applies behaviour change science to develop an intervention to train professionals in motivational interaction behaviours.

**Methods:**

A four-step mixed-methods design included a needs assessment questionnaire (*n* = 203), and pre-test acceptability questionnaires and group interviews (*n* = 15). Systematic intervention development involved: 1) identifying target behaviours, determinants and behaviour change strategies, 2) formulating a theory of change, 3) co-development of intervention materials, and 4) acceptability evaluation. Qualitative data were content analysed and means and medians of quantitative data (58 items, scale 1–5) were explored.

**Results:**

1) We selected nine motivational interaction behaviours (e.g. open questions, clarifying expectations) alongside seven determinants (e.g. self-efficacy) and intervention strategies. 2) Habit and control theories informed self-monitoring, action and coping planning, and habit formation/disruption activities. The intervention was delivered via 3) an online platform, slide shows, videos, and homework. 4) Overall acceptability was high after iterative optimisation. Prior to optimisation, self-insight maps had low overall acceptability (M = 2.8). Implementation intentions were rated acceptable (M = 3.6), but qualitative data revealed substantial misunderstandings concerning intervention coherence, perceived effectiveness, and self-efficacy.

**Conclusion:**

Quantitative-qualitative divergence in implementation intention data underlines the value of mixed-methods evaluation. Habit theory-based exercises show promise, but feasibility and effectiveness testing are needed before conclusions about behavioural impact.

## Introduction

In health and human service professions, it is vital to encounter clients and patients with motivational, supportive, and empowering interaction. According to self-determination theory (SDT; Ryan & Deci, 2017), autonomous motivation and well-being result from basic psychological need satisfaction of autonomy, competence, and relatedness (Ryan & Deci, 2017; Teixeira et al., 2020).

Need-supportive motivational interaction is characterised by autonomy support and structure (Aelterman et al., 2019). When professionals support autonomy, they for example offer choice, provide meaningful rationale, and acknowledge differing views. When professionals provide structure, they, for instance, clarify goals, support action planning, and adjust challenges to be sufficiently challenging, thus supporting the need for competence. Such interaction promotes high-quality motivation (Haerens et al., 2018), sustained health behaviours, including physical activity (PA) in low-active individuals (Ng et al., 2012), and reduced drop-out from sports (Quested et al., 2013). In contrast, chaotic and controlling need-thwarting interaction (Aelterman et al., 2019) is connected with decreased motivation, increased disengagement, and ill-being (Vansteenkiste et al., 2020).

Drawing from the SDT-based circumplex model of (de)motivating styles (Aelterman et al., 2019; Ryan & Deci, 2017), we define professionals' motivational interaction as goal-oriented communication, designed to strengthen motivation for and engagement in behaviour (change), emphasising collaboration, within an atmosphere of acceptance and compassion.

## Applying behaviour change science into motivational interaction

While (de)motivational interaction can often be successfully changed (Raabe et al., 2019), meta-analyses show that many trained professionals do not reach optimal interaction despite comprehensive training (Schwalbe et al., 2014). Furthermore, contextual pressures including workload, established interaction habits, and organisational constraints can undermine the adoption of new behaviours in daily practice (Hancox et al., 2017; Brockbank & Atkinson, 2026; Renko et al., 2025). Effective motivational interaction interventions share key features such as a focus on multiple interaction strategies, multimodal content-delivery (Su & Reeve, 2011) and skill-based activities (e.g. role play in Aelterman et al., 2014). However, there are gaps in the knowledge about how to train professionals effectively. For example, studies fail to report training procedures in detail in ways that would allow them to be tested: evaluations typically report overall effects without detailing which components were expected to produce which changes, or why (Barwick et al., 2012; Madson et al., 2018). Reporting frameworks developed precisely for this purpose, such as TIDieR (Hoffmann et al., 2014) for intervention description and the BCT Taxonomy for labelling the active ingredients for change (Michie et al., 2013), are now standard in behaviour change intervention research. Yet this level of systematic documentation has not been adopted in motivational interaction training: some motivational interaction interventions explicitly have considered behavioural determinants such as acceptability and feasibility beliefs (e.g. Aelterman et al., 2014; Reeve & Cheon, 2016), but none has reported BCT use. Better reporting of theoretical assumptions and intervention components would allow more meaningful evaluation of why effects were modest.

One potential solution to improve training effects is to apply behaviour change science when attempting to change motivational interaction. A behaviour change science perspective requires considering carefully what actions would lead to desired outcomes, and how and why that would happen (see e.g. Hankonen & Hardeman, 2020; Michie et al., 2011; O’Cathain et al., 2019). Based on these considerations, intervention-specific theories of change can be formed, i.e. practical working models that specify the rationale by which changes in behaviour are expected to occur (Davidoff et al., 2015), including theoretical assumptions of intervention mechanisms and behaviour change process. In the present context, behaviour change science serves as an umbrella concept that draws on formal theories to specify what to target and how (Davidoff et al., 2015). The key theories informing the theory of change are SDT, habit theory, and control theory (i.e. self-regulation; Carver & Scheier, 1982) with insights from complexity science.

### Self-determination theory and human motivation

In addition to defining motivational interaction, SDT offers an overarching understanding of people as autonomous, goal-pursuing agents whose motivation is cultivated through experiences of autonomy, competence, and relatedness (Ryan & Deci, 2017). This applies in two ways: it informs how professionals are understood as learners developing their own practice, and it provides the conceptual language through which they support motivation in those they work with. Need-support is thus not only the target of the intervention but also crucial in its delivery.

### Habit theory and self-regulation

Habit and control theories address how behaviour change occurs. Interacting with others involves conscious deliberation and consideration, but it also often unfolds as fast responses to contextual cues, meaning that constant deliberate decision-making in every moment is unrealistic. As such, interaction behaviours can plausibly be conceptualised partly as habits, although this framing has not previously been applied to motivational interaction. Habits are mental associations between contextual cues and behaviours, formed through consistent repetition until the behaviour is triggered automatically (Gardner et al., 2016). For instance, if reflective listening is repeated consistently when encountering a specific cue, it can become a habit, reducing the need for conscious initiation even if the behaviour itself remains consciously performed (Gardner et al., 2016).

Self-regulation (Carver & Scheier, 1982) strategies are key in forming and breaking habits: for example, goal setting, action planning, and self-monitoring support the consistent repetition of a behaviour in the presence of a cue that habit formation requires (Potthoff et al., 2017). Implementation intentions (if-then planning; Gollwitzer, 1999) are one specific strategy that can be applied to habit formation by specifying in advance the cue that will trigger the wanted behaviour (Potthoff et al., 2022). Implementation intentions have demonstrated effectiveness across a range of behaviours, including communication (Gardner et al., 2020). Whilst not all interaction behaviours are suitable candidates for automatisation because some require ongoing deliberation and contextual judgement, self-regulation strategies remain relevant in translating intentions into behaviours (i.e. intention-behaviour gap; Sheeran, 2002). To our knowledge, the use of implementation intentions for interaction behaviour change has been reported only once, and it was associated with increases in the target behaviour (Friedman & Ronen, 2015). As to our knowledge, no prior study has explicitly applied habit theory to motivational interaction; the present study documents this novel application in detail to enable future empirical testing of its effectiveness.

### Complexity science perspectives

The theories above recognise the complexity of behaviours, but do not fully address how to consider participants' unique contexts and social environments. Calls have been made to develop and study interventions with a complexity science perspective (e.g. Hawe, 2015; Heino et al., 2021), and recognise that individuals and interventions are embedded in broader, interconnected networks and that behaviour change is not linear and small changes can produce large effects and unpredictable outcomes (Erdi, 2008). In practice, this means supporting participants in their personalised change paths rather than following a uniform programme.

### Current study

The research on training need-supportive styles to PE and sports professionals remains limited due to relatively low dose (Su & Reeve, 2011) and lack of transparent descriptions of both the intervention content and the rationale for including different intervention features (Barwick et al., 2012; Madson et al., 2018). This study addresses these gaps by using a systematic approach to intervention development and applying behaviour change science to optimise two previous and highly acceptable in- and pre-service teacher interaction trainings (Renko et al., 2020, 2022) prior to feasibility and effectiveness testing. By taking into account the behaviour change principles in regard to self-regulation and habit formation, and complexity science insights (all novel considerations in Supplement 1), we aim to increase the future effectiveness of the final intervention, while also addressing reported challenges, such as fear of losing control when offering choice, choosing the right words when asking open questions, and allocating time and feedback equally (Renko et al., 2020, 2022).

## Aims

We report the development process and empirical optimisation results (regarding acceptability and engagement) of a behaviour change approach to an intervention to teach motivational interaction behaviours to PE teachers, PA counsellors, physiotherapists, health nurses, and sports coaches, focusing on the key uncertainties identified in the development. This intervention includes only the professional participants as targets, and we do not extend our analysis to their ultimate target groups (students, patients, clients, athletes). The research questions by intervention development tasks are:

Task 1: What is the final set of target motivational interaction behaviours? What are the determinants for these behaviours?

Task 2: What is the tentative theory of change (incl. behaviour change techniques) of the intervention?

Task 3: What are the final theory-based materials and what design principles are relevant for delivering the designed intervention?

Task 4: What are the acceptability, engagement and identified optimisation needs in the MotiStyleSport Intervention?

## Methods

The four-task development process drew from established intervention development frameworks, the behaviour change wheel and intervention mapping (Hankonen & Hardeman, 2020). See Table 1 for key tasks. The development was conducted by a core development team of two doctoral researchers (MPa, MPu) and two seniors (NH, ER), with support from an advisory group of five external researchers (PA, LH, TL, SP, KS), two practitioners from the field, and a stakeholder group of 1–2 representatives from all five professional target groups. Across tasks, acceptability and engagement were considered by regular consultations with stakeholders and other experts in motivational interaction. For detailed reporting, see Supplement 2.

**Table 1.** Outline of the four-task intervention development process (Hankonen & Hardeman, 2020) and related research questions, methods and results per each task.

Task title	Research question(s)	Methods/process	Results
1. Identifying and analysing the problem addressed in behavioural terms and developing intervention objectives	What is the final (set of) target motivational interaction behaviour(s)? What influences the behaviours?	Revision of different motivational interaction taxonomies; mapping against the circumplex of (de) motivating styles and previous training. Literature review complementing Matosic et al. (2016); target group survey about perceived barriers (*n* = 203) thematically analysed and mapped against TDF	Nine target motivational interaction behaviours and their 14 demotivational counterparts (see Figure 1). Seven universal behavioural determinants (see Supplement 7)
2. Identifying intervention mechanisms, content, and delivery mode, including the theory of change	What is the tentative theory of change (including the behaviour change techniques) of the intervention?	Analysis of previous training and BCTs, theories mapped against survey results from Task 1 and corresponding TDF domains; co-creation with behaviour change and motivation science experts	The intervention drew from SDT, RAA and habit theory to utilise self-regulation strategies. The intervention outline is presented in Table 2 and the theory of change is presented in Figure 2. See Supplement 7 for how determinants were targeted and Supplements 5 and 6 for participants' change process and planned use of self-enactable BCTs
3. Developing materials and/or technology	What are the final theory-based materials? What design principles are relevant for delivering the designed intervention?	Co-creation with end-users, expert consultation and feedback, internal testing and optimisation of materials and delivery, design principles aligned with SDT and self-regulation BCTs	Content was delivered on an online platform and Zoom video conference via PowerPoint presentations with mini-lectures, exercises, exemplary videos, and printable homework templates. The design principles were behaviour change mindset, clarity and inviting to engage with.
4. Early, iterative testing of the intervention and empirical optimisation	What are the acceptability, engagement and identified optimisation needs in the pre-test?	TFA-based surveys and focus group interviews with training participants (*n* = 15); and facilitator observations during sessions: exploration of means and medians of quantitative data, deductive content analysis of qualitative data	Overall acceptability was high, detected issues related to if-then plans and self-insight map content. See Supplement 9 for all optimisation needs and actions.

### Task 1. Identifying the target behaviours and determinants to develop intervention objectives

A motivational style can consist of multiple specific motivational interaction behaviours (Teixeira et al., 2020), but *when defining the target behaviours and intervention objectives,* time and resources require focusing on a limited set of targets. Our selection process was informed by our previous trainings, SDT literature, and stakeholder discussions. The previous trainings' seven motivational interaction behaviours (Renko et al., 2020) based on motivational interviewing (Miller & Rollnick, 2012) were compared to the techniques used in SDT-based interventions in health contexts (Teixeira et al., 2020) (Supplement 3). The candidate behaviours from these sources were mapped on the circumplex of (de-)motivating styles (Aelterman et al., 2019) to ensure the inclusion of all aspects of autonomy support and structure. The candidate motivational interaction behaviours were presented to the stakeholders for approval.

*To identify determinants of these behaviours*, the literature was reviewed (Rimpelä, 2020) to complement a previous extensive literature review (Matosic et al., 2016). Additional determinants were discussed amongst the developer team to consider possibilities based on behavioural theories. The determinants were evaluated by the core team for their changeability, along with considerations following the APEASE criteria (Michie et al., 2014). The selection process involved individual review of all candidate determinants by MP and MPu, followed by cross-comparison and discussion with core development team members; disagreements were resolved based on relevant literature and evidence. The determinants the intervention could not realistically affect with the time and resources available were removed.

To understand the target groups' contexts, MP analysed electronic survey responses (teachers *n* = 42, coaches *n* = 29, healthcare professionals *n* = 132), recruited via stakeholder unions' electronic newsletters and social media. Questionnaires are available at https://osf.io/jpn7c/. The following open-ended question was content analysed: ‘*What do you see as your most pressing challenges/targets for development in motivating your client/athlete/student? List three things that you wish to change/get support with.*’ The specific target behaviour for these interactions is not specified, as SDT-based motivating interaction is expected to support basic psychological needs irrespective of the ultimate target behaviour.

### Task 2. Defining the theory of change by identifying intervention mechanisms, content, and delivery mode

Following the state-of-the-art intervention design processes (Moore et al., 2019; Skivington et al., 2021), we articulate the theory of change already in the design phase to define the relationships that should be tested in further trials. From our previous training, we included and modified the best parts to teach motivational interaction behaviours based on SDT and Motivational Interviewing (Miller & Rollnick, 2012). We used the Task 1 survey results and existing literature about motivational interaction training to identify applicable TDF domains and conceptually relevant behaviour change theories to outline the BCTs to target behavioural determinants. After drafting the initial theory of change and first drafts of the content, they were discussed and further developed with 3–5 researchers outside the intervention development team who had expertise in counselling, motivation theories, behaviour change science, and complexity science, with a focus on the ideal dose, delivery, and BCTs used. Likely impacts on key determinants were discussed.

### Task 3. Developing materials and/or technology

Material production was done in close cooperation with end-users to ensure acceptability. The core team modified the existing materials from the previous trainings and produced new materials (lectures, exercises, homework templates) for novel components. Stakeholders and motivational interaction experts commented and tested materials throughout the production. Refinements were made according to feedback and disagreements solved through discussions.

### Task 4. Early, iterative testing of the intervention and empirical optimisation

Early testing of the MotiStyleSport intervention was conducted online in November–December 2021 to explore the acceptability of novel components. Participants (*n* = 15, 14 women, 1 man) were recruited through stakeholders, events, social media posts and word-of-mouth. The sample size reflected the resources available for a single pre-test group, which is consistent with the exploratory purpose of this phase. To be eligible, the participants had to currently work as a PE teacher, PA counsellor, physiotherapist, health nurse or sports coach. Recruitment was halted after reaching the target of 15 eligible participants. Participants were 8 physiotherapists, 3 sports coaches, 2 PA instructors, 1 PE teacher, and 1 multiple professions. Participants enrolled through an online form and provided informed consent. For each intervention session, the participation rate was 87% or above. One participant dropped out after the half point of the intervention.

The intervention consisted of six two-hour online sessions (12 hours in total) over 6 weeks and was facilitated by MPu (licensed psychologist) on Zoom. MPa assisted. The sessions included mini-lectures, discussions, and small group tasks/discussions in breakout rooms. Outside the sessions, the participants had one peer-support meeting and homework after each session they could tailor to fit their needs. See Table 1 for more details.

The data included field notes, and questionnaire and interview data collected after training sessions 2–6. Immediately post-sessions, participants completed *short online questionnaire*s based on the theoretical framework of acceptability (TFA) to report their experienced acceptability (6 dimensions, ethicality excluded as unlikely to be a concern in a voluntary professional training focused on motivational interaction) (Sekhon et al., 2017) of selected components (10–14 items per session) and the whole training (28 items) on a scale from 1 (completely disagree) to 5 (completely agree). For example, ‘I liked the mini-lecture on if-then plans’. Items mostly aligned with the recent items by Sekhon et al. (2022).

To gather more in-depth comments regarding the acceptability of the novel components, all participants participated in one of five *semi-structured focus group interviews*, 2–3 participants at a time via Zoom (duration 20–25 minutes), interviewed by MPa. The questions were formulated to elicit acceptability-related commentary on the intervention content and they were reviewed by ER, experienced in TFA-based qualitative data collection. Recordings were transcribed verbatim. Data collection materials are available at https://osf.io/9nvx7/.

Facilitators categorised field notes under identified challenges in post-session facilitator debriefs. MPa calculated the means and medians of quantitative survey data for each acceptability dimension for the novel intervention components. Overall acceptability scores for the self-insight map and implementation intention assignments were calculated as the mean of the assessed acceptability dimensions per assignment. MPa content analysed responses to the open-ended questions in the survey. ER conducted a deductive qualitative content analysis of the interview data, guided by the dimensions of the TFA (Sekhon et al., 2017). The analysis followed the general phases of qualitative content analysis (Elo & Kyngäs, 2008). In the preparation phase, ER read the interview transcripts in their entirety to gain an overall understanding of the data. Next, ER read through the transcripts several times and made preliminary notes on how the participants' accounts related to the predefined TFA dimensions. ER then used the TFA dimensions as a categorisation matrix and reviewed all the data for content, systematically coding segments of text according to the predefined categories. The first draft of the categorisation was reviewed with the co-authors to determine whether the coded extracts fit the TFA categories. There were no major disagreements. As this study constitutes a pre-test phase focused on acceptability and optimisation, behavioural measures were not included by design.

### Ethics statement

Study procedures received a favourable review from the Research Ethics Committee in the Humanities and Social and Behavioural Sciences in Helsinki (10/2021, 58/2021).

## Results

### Task 1

The selection process resulted in nine target motivational interaction behaviours: six autonomy-supportive and three structuring, and their demotivating counterparts, resulting in 14 behaviours to limit, avoid, or unlearn (Figure 1). The stakeholders approved these without concerns. The intervention focused on developing motivational interaction behaviours without specifying the behaviours these motivational interaction behaviours are targeting. This choice was theoretically informed as SDT-based research has shown that a motivating style supports basic psychological needs irrespective of ultimate target behaviours.

**Figure 1.**
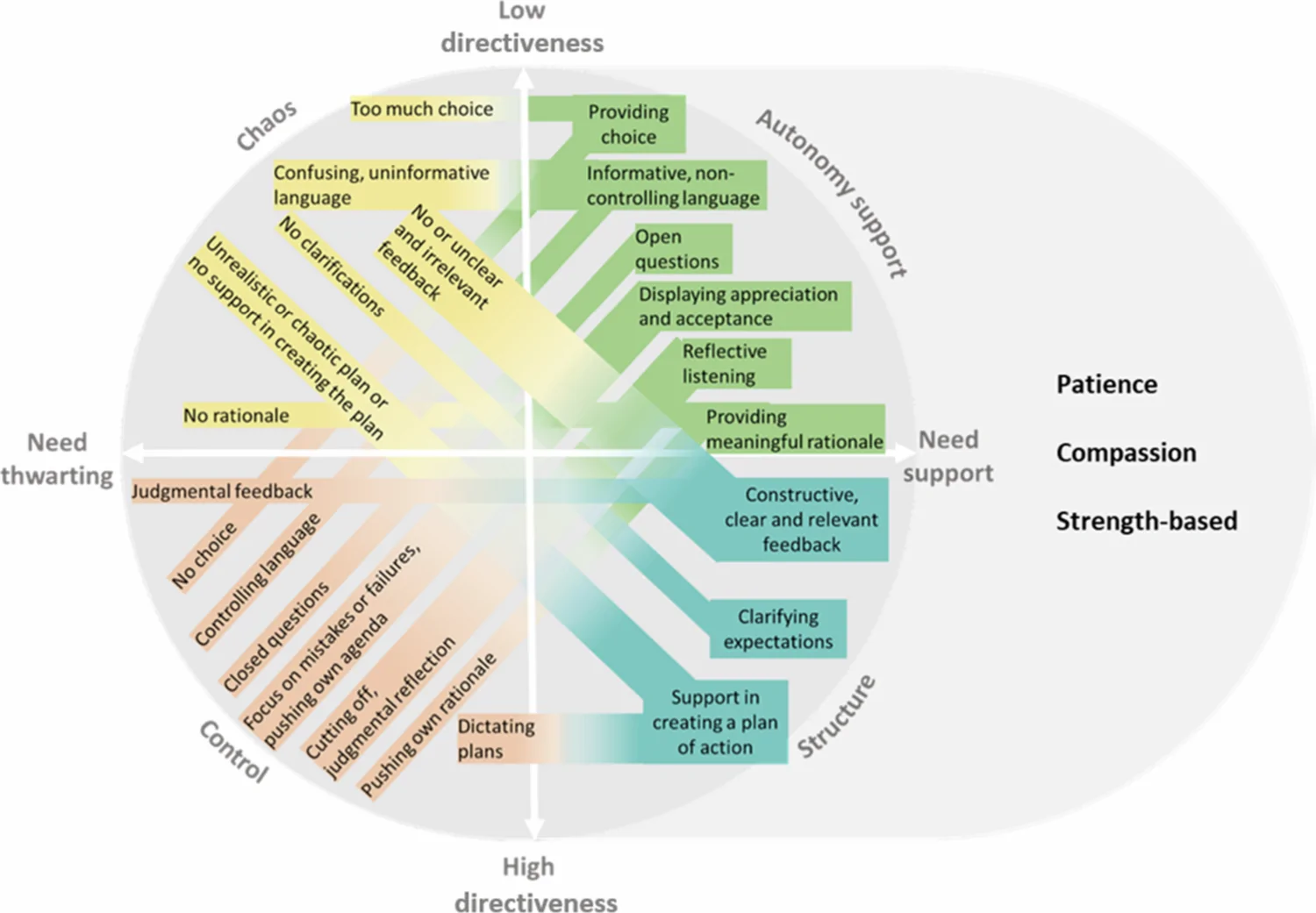
The selected target behaviours for the MSS intervention: 9 motivational interaction behaviours with their de-motivational counterparts mapped onto the four main sectors (autonomy support, structure, control and chaos) of the circumplex of (de)motivational styles by Aelterman et al. (2019).

The literature review (Rimpelä, 2020) identified 27 new relevant publications and 23 new determinants such as social unity and achievement of goals, in addition to those identified by Matosic et al. (2016) (Supplement 4). Reflecting on habit theories, the developer team considered four additional determinants (knowledge, skills and capabilities, self-efficacy, cues) to support habit formation, resulting in 54 candidate determinants. Determinants were excluded as direct targets if they were considered largely stable background characteristics (e.g. personality traits, causality orientations), structural or organisational factors beyond the scope of a training intervention that were instead acknowledged as contextual conditions within which participants work (e.g. time constraints, administrative pressures), skills better taught within the intervention as behaviour change strategies rather than targeted as determinants (e.g. emotion regulation), or distal outcomes of the intervention rather than proximal mechanisms (e.g. need satisfaction of clients). The final key determinants considered changeable, targetable and compatible with SDT, habit theory and control theory assumptions, were outcome expectations, norms, self-efficacy, skills (to use motivational interaction, to self-regulate and to regulate emotions), autonomous motivation, knowledge and self-insight of one's motivational interaction behaviours.

In addition to universal determinants, determinants were identified for different motivational interaction behaviours to highlight their unique features. For example, reflective listening requires skills to provide summaries and recognise as well as verbalise others' feelings, whereas providing a meaningful rationale requires understanding what could be meaningful for the other person/group and why.

The most often mentioned barriers to motivational interaction behaviours in the surveys were for *teachers*, lack of time, uninterested/unmotivated students, and big groups, for *healthcare professionals*, lack of time, lack of knowledge about options for health behaviours, and creating a meaningful relationship with the clients, and for *coaches*, lack of time, motivating the athletes, and equal treatment of athletes.

The final intervention objective was *to teach participants a basic understanding of how and why to use motivational interaction behaviours* (educational objective), *and to support change in participants' motivating styles, i.e. decrease chaotic and controlling styles and increase autonomy supportive and structuring styles as well as instil a compassionate, patient and strength-centred general philosophy of supporting people and to sustain these changes over the long-term* (behaviour change objective).

### Task 2

For the theory of change, we conceptualised the change to operate on two different levels: 1) at the *general level* as a motivation to pursue one's motivational interaction behaviours, and 2) at a *personal level* through individualised behavioural practice of the target behaviours in the participants' unique contexts. That is, the intervention recognised the participants' role in identifying and prioritising their personal determinants and barriers (Figure 2). The prioritisation of the motivational interaction behaviours to practice was explicitly left to the participants and they were not expected to automatise all of them. The participants were supported in developing sufficient understanding of their situation/needs during the self-monitoring exercises. A visual depiction of participants' behaviour change process was created and displayed throughout the training (Supplement 5). It was formulated as a bridge over the intention-behaviour gap (Sheeran, 2002), the steps representing self-enactable BCTs to change behaviours (Supplement 6).

**Figure 2.**
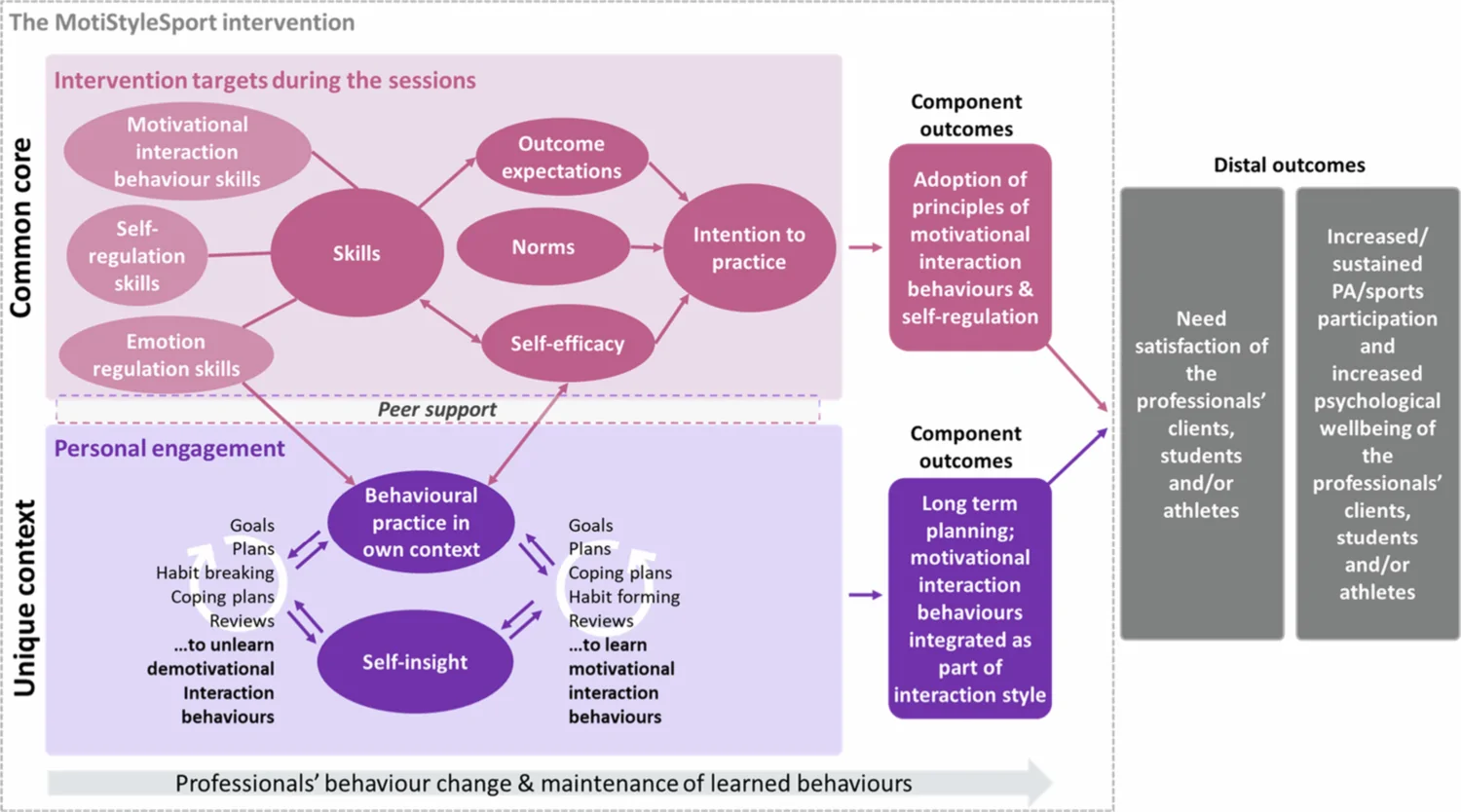
Theory of change in MotiStyleSport. The intervention consists of two main components: 1) the common core for all participants resulting in the adoption of the principles of motivational interaction behaviours, self-regulation, and emotion regulation skills 2) the unique contexts in which each participant chose to engage with the intervention activities resulting in the long-term maintenance of motivational interaction behaviours. In the common core, the intervention targeted outcome expectations, norms and self-efficacy in attempts to build participants' intention to practise the motivational interaction behaviours. Motivational interaction behaviours and self-regulation techniques were practised also, as they are thought to affect the positive outcome expectations and build self-efficacy to use the motivational interaction behaviours and to use self-regulation skills. In participants' unique contexts, participants practised the skills and gathered more insights into their personal settings through the self-regulation cycle of goal setting, planning, habit breaking, coping plans and reviewing (and adjusting) goals and plans. As distal outcomes, the professionals' clients, students and/or athletes should have enhanced basic need satisfaction which in turn should lead to enhanced PA and reduced drop-out from sports once the professionals adopt and maintain the motivational interaction behaviours in daily practice.

The novel intervention theory paid special attention to aiding participants to optimally adopt the target behaviours. This content was informed by SDT, self-regulation literature (Carver & Scheier, 1982), habit theory (Gardner & Lally, 2018; Potthoff et al., 2022) and complexity principles (Hawe, 2015). The reasoned action approach (Fishbein & Ajzen, 2011) was reflected upon when building conscious intention to modify one's motivational interaction behaviours (i.e. change people's norm beliefs, outcome expectations, and self-efficacy).

In terms of intervention delivery and content, the intervention's session outline is presented in Table 2 (more detailed operationalisations of BCTs in Supplement 7). Several BCTs appear across multiple sessions because the target behaviours were new to participants and required repeated instruction and consolidation, and because self-regulation was central to the behaviour change process. The circumplex of (de)motivating styles (Aelterman et al., 2019) was used to present the programme outline and target behaviours, as its sectors provide a clear structure and enable covering also demotivational interaction behaviours.

**Table 2.** MotiStyleSport session outline and main behaviour change content.

Session	Motivational interaction behaviours	Learning outcomes regarding behaviour change strategies and skills	Determinants targeted	BCTs delivered (BCTT v.1, Michie et al., 2013)
Session 1: Introduction	Minilectures and discussions on: **‐** basics of motivation, goals and motivational interaction behaviours (SDT; circumplex) **‐**Two autonomy-supportive behaviours: **‐** Providing a meaningful rationale **‐** Non-controlling informative language	During the session, the participants: **‐** Understand the interaction behaviour change process phases/elements with the help of the behaviour change bridge. After the session, the participants, with the help of a self-monitoring sheet **‐** Self-monitor 1–2 motivational interaction behaviours and their de-motivational counterparts **‐** Self-monitor consequences of the behaviours	Knowledge Skills Autonomous motivation Outcome expectations Self-efficacy	1.4 Action planning 2.3 Self-monitoring of behaviour 2.4 Self-monitoring of outcome(s) of behaviour 4.1 Instruction on how to perform the behaviour 6.1 Demonstration of the behaviour 8.1 Behavioural practice/rehearsal 8.7 Graded tasks 9.1 Credible source 15.3 Focus on past success
Session 2: Autonomy support	Minilectures, discussions, and rehearsals of: **‐** Open questions **‐** Reflective listening, **‐**Showing appreciation and acceptance, **‐** Providing choice	During the session, the participants: **‐** Understand what could influence one's interaction behaviours **‐** Explore what influences them by creating a self-insight map of things that aid or hinder using a previously self-monitored motivational interaction behaviour and its de-motivating counterpart(s). After the session, the participants, with the help of a planning template (incl. coping planning for execution) **‐** Execute a planned behavioural experiment	Knowledge Skills Autonomous motivation Outcome expectations Self-efficacy Self-insight	1.2 Problem solving 1.4 Action planning 4.1 Instruction on how to perform the behaviour 4.2 Information about antecedents 4.4 Behavioural experiments 6.1 Demonstration of the behaviour 8.1 Behavioural practice/rehearsal 8.7 Graded tasks 9.1 Credible source 15.3 Focus on past success
Session 3: Structure	Minilectures, discussions, and rehearsals of: **‐** Constructive, clear and relevant feedback **‐** Clarification of expectations **‐** Support in creating a concrete plan of action	During the session, participants: **‐** Understand about basics of habit formation and breaking **‐** Understand about of basics of cue-behaviour associations and if-then plans **‐** Create one if-then plan for forming/strengthening a wanted habit (use of motivating technique) and one for breaking an unwanted habit (use of de-motivating technique). After the session, the participants: **‐** Execute the if-then plans in one's own context	Knowledge Skills Autonomous motivation Outcome expectations Self-efficacy Self-insight	1.5 Review behaviour goal(s) 4.1 Instruction on how to perform the behaviour 4.4 Behavioural experiments 6.1 Demonstration of the behaviour 7.1 Prompts/cues8.3 Habit formation 8.1 Behavioural practice/rehearsal 9.1 Credible source
Session 4: Combining motivational interaction behaviours	Minilectures, discussions, and rehearsals of: **‐** How to use combinations of different motivational interaction behaviours **‐** When encountering resistance or conflicts **‐** In advising without pressing **‐** In inviting change talk	During the session, the participants: **‐** Understand the difference between emotion regulation vs. suppression in interaction situations **‐** Create an emotion regulation If-then plan (negative emotion as cue). After the session, the participants, with the help of planning templates **‐** Plan for detecting and reacting to change talk **‐** Execute the chance talk plan (at least detection) **‐** Make a coping plan for when encountering resistance or conflicts	Knowledge Skills Autonomous motivation Outcome expectations Self-efficacy Self-insight Norms	1.1 Goal setting (behaviour) 1.2 Problem solving 1.4 Action planning 4.1 Instruction on how to perform the behaviour 4.2 Information about antecedents 5.3 Information about social and environmental consequences 6.1 Demonstration of the behaviour 6.2 Social comparison 6.3 Information about others' approval 7.1 Prompts/cues 8.1 Behavioural practice/rehearsal 8.3 Habit formation 8.7 Graded tasks
Session 5: Challenging situations	Minilectures, discussions, and rehearsals of: **‐** Motivational interaction behaviours in challenging situations **‐** Ways to overcome challenges in learning and implementing the motivational interaction behaviours	During the session, the participants: **‐** Create one's own self-insight map of one motivational interaction behaviour, including factors that facilitate and hinder its use and plans to strengthen the facilitators and cope with the hindering factors. After the session, the participants: **‐** With the help of the self-insight map, follow interaction situations and complete the self-insight map **‐** Meet for a peer support session (2-4 participants) to discuss how practising and using the techniques have gone so far	Knowledge Skills Autonomous motivation Outcome expectations Self-efficacy Self-insight Norms	1.2 Problem solving 1.4 Action planning 1.5 Review behaviour goal(s) 3.1 Social support (unspecified) 4.1 Instruction on how to perform the behaviour 4.2 Information about antecedents 5.3 Information about social and environmental consequences 6.1 Demonstration of the behaviour 6.2 Social comparison 6.3 Information about others' approval 8.1. Behavioural practice/rehearsal
Session 6: Recap	Minilectures, discussions, and rehearsals of: **‐** to integrate all that has been learned into a coherent set **‐** ways to implement behaviours into daily work	During the session, the participants: **‐** Learn about self-motivational techniques to maintain their motivation for long-term behaviour change **‐** Learn about ways to seek social support for learning and maintaining new behaviours	Knowledge Skills Autonomous motivation Outcome expectations Self-efficacy Self-insight Norms	1.1 Goal setting (behaviour) 3.1 Social support (unspecified) 4.2 Information about antecedents 5.3 Information about social and environmental consequences 6.3 Information about others' approval 8.1 Behavioural practice/rehearsal

Activities and examples were adapted from the previous training (Renko et al., 2020) to suit all professions, based on professionals' survey responses.

As a novel approach to learning motivational interaction behaviours, we embedded habit-based BCTs to promote the real-world use of motivational interaction behaviours. Habit theory informed the habit forming and breaking activities aiming at long-term maintenance of motivational interaction behaviours by helping the participants identify behavioural cues in interaction situations and creating if-then plans for habit building (Hagger et al., 2016; Potthoff et al., 2017). We utilised self-monitoring and self-insight maps to support participants with identifying personal cues that trigger wanted/unwanted motivational interaction behaviours. These included not only individual-level triggers such as emotional state, but also contextual and organisational factors such as time constraints or collegial pressure. Participants were trained to create their own if-then plans for habit formation/breaking by separating between stable and predictable structural and unpredictable but common events in interaction and pairing them with motivational interaction behaviours (Table 3).

**Table 3.** Example of application of habit theory and if-then plans to form habits in the MotiStyleSport intervention.

Type of cue	Relevant motivational interaction behaviours	Example of an if-then plan
Stable and predictable structural event in interaction (e.g. start of the session, end of an activity)	**‐** Clarifying expectations **‐** Providing constructive, clear and relevant feedback **‐** Providing choice	When the session starts, then I clarify the expectations for the session.
Unpredictable yet common event in interaction (e.g. students saying they do not want to do an activity, athlete getting upset after performance, one’s own internal impulse)	**‐** Non-controlling, informative language **‐** Reflective listening **‐** Open questions **‐** Displaying appreciation and acceptance **‐** Providing a meaningful rationale + taking reflection space before acting	If my client shows hesitation (e.g. I would like to… but…), ‘then I use reflective listening (e.g. on one hand… on the other…).’ If I am about to say ‘you have to…’, then I stop myself, and say ‘we could…’.

Following SDT, the intervention content and facilitation was designed to support participants' active agency and autonomy in behaviour change. Complexity principles were used to consider participants' individual change paths. They were guided in creating their own self-insight maps to foster self-regulation by identifying opportunities for change by detecting relevant determinants for one's behaviours. The participants met in pre-assigned peer groups to discuss their learning experience after the fifth session with the help of homework templates and self-insight maps. They could freely choose the format of the peer group (e.g. online meeting, call, texting).

### Task 3

The intervention materials consisted of presentations with mini-lectures, exercises, exemplary videos, and printable homework templates. Participants downloaded all materials from an online platform Moodle. Three key design principles were followed. Promoting the *behaviour change mindset* principle, all content approached interaction as a changeable behaviour, and materials were designed to have elements that support participants' behaviour change (e.g. tips, examples, coping plans). C*larity* was promoted by systematic colour coding and use of icons for target behaviours, design indications (e.g. pen and thinking icons) for homework assignments, gradual animations, illustrations and clear fonts (Corbel & Calibri, point size above 16). The principle *inviting to engage with* was promoted by using a consistent colour palette of blue, green, orange and red and illustrative gender-neutral human figures for interaction examples.

### Task 4

The findings are interpreted as preliminary evidence informing optimisation rather than as definitive conclusions about the intervention's acceptability which is more rigorously evaluated in the subsequent feasibility study (Puolamäki et al., 2025). Overall, high acceptability is indicated (means and medians 3 or higher, unless reversed) for the training and most components. The key quantitative figures per intervention elements (*n* = 8–13) are presented in Supplement 8. The number of responses varied because not all participants completed the post-session survey after every session. Here, we highlight the detected issues.

In the quantitative evaluations, the first version of the self-insight map assignment received the lowest acceptability ratings (overall acceptability M = 2.8; Mdn = 2.9) but the ratings improved in the next iteration in session 4 after substantial changes (M = 3.8, Mdn = 3.9), where participants were given a limited set of target behaviours to choose from, more step-by-step instructions with examples and time to work individually before group discussion. Implementation intention content was rated acceptable (M = 3.6, Mdn = 3.5) but facilitator observations suggested partial misunderstanding, as participants' if-then plans did not include a motivational interaction behaviour (e.g. If I need to concentrate, I close my office door). Acceptability dimensions assessed for the overall means included affective attitude, burden, intervention coherence, perceived effectiveness, and self-efficacy; opportunity costs were additionally assessed in the second iteration for implementation intentions. These components illustrate how quantitative and qualitative data complemented each other in different ways: for the self-insight map, interview findings corroborated the low ratings and elaborated the reasons behind them, whereas for implementation intentions, the interviews revealed substantial problems that the high quantitative ratings alone had obscured. The qualitative findings are presented below.

### Self-insight map v1

The barriers identified in the interviews related to the acceptability dimensions of intervention coherence and self-efficacy, and concerned time management, understanding the instructions, and providing structure and rationale. The tight schedule completing the map in time seem impossible (TFA-Self-Efficacy), some said that they would have appreciated a moment to draw in private before small group discussions where one is expected to discuss and not to draw. ‘*We did not get to the drawing at all either -- so maybe to have a little private moment to draw because the whole group just starts talking then’*

Challenges in understanding instructions were reported leading to chaotic and confused feelings (TFA-Self-Efficacy) and made it difficult to choose where to focus (TFA-Intervention Coherence). Some pointed out that briefing could have taken place in several stages throughout the assignment.

Some experienced that rationale and structure were not clearly provided, and it was difficult to make choices and understand the purpose of the task (TFA-Intervention Coherence). ‘*Maybe a clear instruction like, take one situation into focus.’, ‘Maybe some kind of little friendly blackmail that it is beneficial to do the first part because we need it in the second – a more detailed rationale.’*

However, the interviewees viewed the assignment's potential and thought that it could be useful in understanding factors that underlie interaction situations (TFA-Perceived Effectiveness). ‘*This is a really good activity to think what are the things in the background and these guiding questions were really good but in the beginning it took some time to grasp what (the idea is)’*

### If-then plans

Despite the high quantitative ratings, the interviews revealed acceptability issues. The barriers concerned understanding the instructions and abstract terms (TFA-Intervention Coherence), the rationale became clear only after completing the task (TFA-Perceived Effectiveness). These challenges were reported to lead to uncertain and confused feelings (TFA-Self-Efficacy).

Some experienced that adding time and providing examples could have made the assignment clearer. ‘*I scribbled something a little and maybe in the right direction but there could be some examples’ ‘You also have a very tight schedule and I would want to have some looseness. In recaps you can use the chat too, write your own examples there and maybe some light-bulbs would turn on’.*

However, the interviewees saw the assignment potentially useful in identifying habitual interaction behaviours and reflecting ways to change them (TFA-Perceived Effectiveness). ‘*It specifically helped in recognising your own behaviour in situations and how to change it.’*

### Participant engagement with the intervention content

A crucial engagement issue was the non-completion of homework. Participants reported not having time between sessions and some of the homework looking too heavy with the instruction texts. Furthermore, the templates were online and participants had to print them out themselves. ‘*It is necessary to have it on paper – also there is a lot of text and content and I tend to open it on my phone and zoom and look around and it is not as handy’.*

As a solution, in later sessions, we reserved time to start the homework during the sessions. This was perceived as an effective way to carry out the homework: ‘*Good that there has been that kind of systematicity that we have gone through together and started to plan the homework a little which makes it more concrete and easier to execute’.* Also, having options for homework was perceived positively as participants could choose those that best served their situation and goals, making the task more enjoyable. ‘*Having options for the homework creates a positive feeling about getting to choose what to do. It is much nicer to do the homework when you can choose the best-suited options’.*

Inviting participants by name to speak was perceived as a good way to engage participants in an online environment: ‘*I like that you give the participants turns to speak. I am shy speaking out loud or commenting on Zoom even when I have ideas or something to say. It is nice to be given the floor.’*

The social aspect of the course was perceived as engaging. Session activities and group discussions were perceived as a good way to learn as they offer a chance to try the behaviours with guidance and a chance to learn from others'. A peer support system was suggested in the interview unprompted before peer groups had been carried out: ‘*Could it be possible to do the homework with someone so that there would be like a pair in this group. – You could do the assignment and then discuss it for a little while’*. After having a peer support session, being accountable to another person was perceived helpful in completing the homework. ‘*I really liked having a pair. It gives me a boost to do and plan the assignment better – writing the message gathered my thoughts about the situation where I practised. You don't think about it the same way alone as you do when you explain it to someone else.’*

### Optimisation needs and actions

Optimisation needs were identified based on facilitator observations and field notes, surveys and focus group interviews (Supplement 9). Key optimisation needs related to 1) restructuring the presentation of motivational interaction behaviours for clarity, 2) loosening the schedule, 3) simplifying the self-insight map and 4) the habit content, 5) adding more peer support, 6) adding one more week between sessions for homework supported by a physical workbook to support engagement. The changes to the self-insight map and if-then plan components were the most extensive, involving redesign of instructions, templates, and task structure, as well as internal testing before the feasibility study. All detected optimisation needs were implemented in the feasibility study conducted later (Puolamäki et al., 2025).

## Discussion

We presented the development and empirical optimisation of the MotiStyleSport online training for sports and PA professionals in motivational interaction behaviours. The intervention was considered highly acceptable in qualitative and quantitative evaluations, similarly to its predecessor (Renko et al., 2020). The novel parts of the intervention related to explicitly defining interaction as a behaviour, revision of the target motivational interaction behaviours and the behaviour change content, specifically the different habit strategies. However, the novel components of self-insight maps and implementation intentions had acceptability issues, illustrating that excessive conceptual complexity can reduce usability, and careful operationalisation is therefore essential even for theoretically well-grounded components. These issues were taken into account in the next iteration, and the acceptability improved. Prior to the feasibility study (Puolamäki et al., 2025), both components were substantially rebuilt.

This study brings novelty value to the literature in various areas. First, it focuses on behaviour change and maintenance of self-regulation strategies in the context of motivational interaction behaviour change by explicitly specifying target behaviours and their determinants (RQ 1) and implementing a theory of change grounded in behaviour change science (RQ 2). That is, to our knowledge, this study is among the first to conceptualise (de)motivating styles as behaviours and empirically investigate behaviour change strategies in the participants' change processes. Even if elements of this approach may have been applied elsewhere, it has not been reported in this level of detail in regard to interaction behaviours. The overall approach conceptualised not only the target behaviours, but their counterparts, aiding participants to recognise different aspects of their motivational interaction behaviours. Particular attention was given to habit formation strategies, self-insight and mapping of one's interaction situation as a system. Specifically, habit forming and breaking skills considering participants' unique motivational interaction behaviour change processes are introduced to form and sustain new beneficial interaction routines. However, the pre-test raised the possibility that the six-week timeframe and weekly session intervals may not provide sufficient time for participants to practise and consolidate new behaviours between sessions. This was addressed in the feasibility study by doubling the intervals between sessions (Puolamäki et al., 2025), allowing more time for real-world habit formation. Future research could focus more closely on what cues are most effective in supporting real-world habitual behaviour and why: do participants more readily form habits in response to stable/predictable cues (e.g. start of a session) versus more unpredictable cues that commonly occur (e.g. athlete getting upset after poor performance)? This distinction (stable vs. unpredictable but common) would allow investigating whether forming habits around stable, predictable cues might facilitate the adoption of more reflective motivational interaction behaviours in less predictable contexts, where conscious deliberation remains necessary but may be supported by the automatisation of simpler, foundational interaction responses.

Second, it is taking initial steps towards using insights from complexity science to complement traditional approaches (see Heino et al., 2021) by acknowledging participants' individual change paths and wider social context where interaction behaviours are embedded (RQ 2). In line with the complex systems approach, participants were encouraged to self-set goals that fit their own context, recognise unique influences via self-insight maps, and create sustainable changes by engaging their social environment in the change process (e.g. inviting feedback, collaborating with peer groups). Rather than reducing motivational behaviours to the individual level or imposing a generic intervention logic model universally on all participants, the intervention acknowledged that professionals' interaction behaviours are shaped by and embedded in their broader social and organisational contexts. We acknowledge that these were applied at the level of design guidance in this pre-test phase, and complexity principles such as attention to feedback loops, critical fluctuations, and attractor dynamics (Heino et al., 2021, 2022) were more comprehensively incorporated in the feasibility study phase.

Third, we report the systematic use of intervention development frameworks and tools, in the context of interaction behaviour change (RQs 1–4) and give intervention acceptability and participant engagement centre stage (RQs 4) in the development process of a motivational interaction behaviour intervention. Our reporting is consistent with current guidance for intervention development studies (Duncan et al., 2020), which calls for transparent documentation of development decisions, theoretical assumptions, and the rationale for intervention components.

As a strength, the inclusion of an early pre-test prior to the feasibility study allowed systematic identification and resolution of implementation problems before larger-scale testing. In many intervention development contexts, a feasibility study represents the first empirical test; the addition of this earlier optimisation phase means the feasibility study can proceed on a more solid foundation. Specifically, gathering brief and immediate feedback after each session provided detailed information on the acceptability of individual contents and enabled iterative optimisation. The combination of quantitative and qualitative data was a further strength, as participants may rate a component positively whilst expressing reservations in interviews, or praise it verbally whilst still rating it low. Having both data sources therefore provided a more complete picture than either alone. Also, considering acceptability together with engagement was informative: the misunderstanding of if-then plans could have gone undetected if only acceptability ratings had been considered, as the participants did not realise their misunderstanding. This divergence between acceptable quantitative ratings and qualitative evidence of misunderstanding illustrates a broader methodological lesson for acceptability work: self-report ratings alone may be insufficient to detect that a component has been misunderstood, and mixed-methods evaluation is therefore valuable whenever acceptability is assessed.

As a limitation, the empirical test sample was relatively small (*n* = 15) reflecting the time and resources available for a single pre-test group. This can raise the question whether additional groups would have yielded different findings. The sample also consisted mostly of women and physiotherapists, despite the intervention targeting multiple professional groups, which leaves a possibility for undetected issues specific to men or other professions – even though there were some participants from all target occupational groups and both genders. Recruitment possibly introduced self-selection bias, attracting individuals already interested in motivational interaction, which may have inflated acceptability ratings. Reporting bias is also possible, due to self-reports and the intervention delivery by the research team. Furthermore, the research team and intervention participants shared a common cultural background, which may have influenced both intervention design and interpretation of findings, and motivational interaction principles may vary across cultural contexts and professional traditions. However, we are not making statistical inferences and use the pre-testing data merely for detecting indications of possible issues in the acceptability of the interventions. These limitations were systematically addressed and further investigated in the feasibility study (Puolamäki et al., 2025), which included a larger and more professionally and demographically diverse sample of 145 participants.

This study provides preliminary evidence that it is conceptually possible and acceptable to use behaviour change principles like habit formation to support the adoption of new motivational interaction behaviours. The circumplex of (de)motivating styles (Aelterman et al., 2019) appeared workable as an organising structure, and its possibilities as a learning tool for motivational interaction behaviours should be explored further. Whether the intervention is feasible to implement at scale, whether it produces actual changes in motivational interaction behaviours, and whether those changes are sustained over time, remains to be established through a feasibility study, longitudinal follow-up, and objective behavioural assessment before conclusions regarding effectiveness can be drawn.

## Supplementary Material

Supplementary_material.docx

## Data Availability

The data that support the findings of this study are available from the PI upon reasonable request.

## Open Scholarship

This article has earned the Center for Open Science badges for Open Materials. The materials are openly accessible at https://osf.io/jpn7c/.and https://osf.io/9nvx7/.
