# Supplementary material for: Applying behaviour change science to change motivational interaction in physical activity and sports professionals: stepwise development and acceptability pre-test of the MotiStyleSport intervention
Source: Health Psychol Behav Med. 2026 Oct 9;14(1):2716945. doi: 10.1080/21642850.2026.2716945 (PMC13656641; doi:10.1080/21642850.2026.2716945)
Supplement: Supplementary file 1 [file RHPB_A_2716945_SM0770.docx]

# Supplements

## Supplement 1. *List of novel considerations taken into account in outlining the intervention to promote basic need supportive motivational interaction behaviours.*

| **Consideration** | **Reasoning** |
| --- | --- |
| Conceptualising interaction styles as a set of behaviours | Conceptualising interaction as behaviours enables utilising behaviour change science principles and tools systematically in the attempts to change motivational interaction |
| Utilising complexity principles in intervention design: | Calls have been made to develop and study interventions with complexity science perspective; emerging evidence that interventions aligned with complex dynamical adaptive systems approaches fare better than those that do not. Even a light incorporation of the following principles should be beneficial in taking steps towards interventions that better take into account the complex nature of behaviours:   1. A complex interconnected system: A multitude of interwoven, interdependent, and interacting variables and timescales. Target behaviour(s) are influenced by various interconnected influences that can be intervened on. Intervention processes are interdependent and interacting. 2. Intervention participants are active, learning agents who self-organize the attempt to change their behaviour. 3. Contextualised processes, speciﬁc to each individual: Individuals follow meaningfully different change trajectories that develop and change with time. 4. Not restricted to linear dynamics: Inputs are not necessarily proportional to outputs, and long periods of apparent stability can precede short periods of rapid change. |
| Utilising habit theory in changing interaction behaviours | Interaction (and reactions) can be automatic; self-regulation of behaviour requires much conscious effort but once habits are formed, they are automatic; self-regulation techniques can be used to create habits |
| Considering intervention acceptability and participant engagement in the development | Intervention participants are active agents; interventions will not work as intended if the participants do not find the intervention acceptable, comprehend the intervention and perform the cognitive and behavioural skills taught in it |
| Delivering the intervention remotely | Enables participation from geographically diverse areas; savings in participation costs; savings in travel time for participants and intervention deliverers |
| Training longer in duration | Longer training enables more chances for instructed behavioural practice; especially important for using habit formation and breaking strategies that require consistent repetition to form cue-behaviour associations |
| Multidisciplinarity | Motivational interaction behaviours’ principles share similarities across different contexts; synergistic benefits when discussing problems from various areas reported |

## Supplement 2. *Stakeholder involvement reported using the GRIPP2 (Staniszewska et al., 2017)*

| **Section and topic** | **Item** |
| --- | --- |
| **1: Aim**  Report the aim of the study | To develop consensus on the key elements (learning objectives, determinants to target, delivered content) of the MotiStyleSport. To collaboratively involve stakeholders as partners at all stages in the development process of MotiStyleSport |
| **2: Methods**  Provide a clear description of the methods used for PPI in the study | We formed a stakeholder group consisting the following to assist at all stages of the development of the MotiStyleSport intervention:   1. An advisory group of five external researchers with extensive backgrounds in need-supportive interaction, motivation, motivational interviewing, psychology, intervention development, and health behaviour change research, 2. Practitioners/professionals from the field (n=2) with background in motivational interaction trainings 3. A group of 1-2 representatives from all five professional target groups (total n=6)     The stakeholders were involved in forming the theory of change, as refining the focus and the content of the intervention and setting the research schedule before the pre-test in workshops separately organised for different groups. In more detail:   - The advisory group commented and discussed a long-list of possible theory-based determinants for behavioural outcomes to form the theory of change - The advisory group and target group representatives discussed and commented on the long-list of behavioural outcomes (i.e., motivational interaction techniques), behaviours to de-implement (i.e., de-motivational interaction techniques) and related sub-behaviours (if any) to refine the focus of the intervention. - Based on the formed theory of change existing research, the advisory group discussed and commented the preliminary set of behaviour change techniques and self-enactable behaviour change techniques (e.g., habit mental rehearsal, guided practice) and agreed on the final set of (self-enactable) behaviour change techniques after a revisions based on comments. - In the content development phase, all stakeholders commented on examples of situations/factors compromising optimal motivational interaction (for different groups) to refine the case examples and contextualising of the training. - In the content testing phase, the newly developed materials were delivered by the facilitator and discussed in a facilitated discussion with the focus on practicability, delivery, understandability, and meeting the key learning. In the testing phase, practical experts checked comprehension of the materials from the lay perspective. In addition to the practical experts, a social psychology student also assisted in testing and reviewing the self-enactable homework. - In planning the research timeline, the target group representatives provided information about their professional groups’ yearly schedules and the activities were planned accordingly. They also participated in the recruiting by advertising the pre-test and sending out registration letters. - The advisory group members significantly contributed to edits of the paper and are coauthors.     Throughout the stakeholder involvement, activities/content not reaching consensus were adapted through a facilitated discussion. |
| **3: Results**  Outcomes— Report the results of PPI in the study, including both positive and negative outcomes | The stakeholders contributed to the study in several ways, including:   - The stakeholders helped in identifying the key behavioural determinants - The stakeholders contributed in refining the intervention focus, key concepts, and activities - Considering the evidence and their experience from the field, the stakeholders highlighted the importance of including real life examples for all expertise groups, and had the final approval for all examples created. - The practical experts helped ensure that the terminology reflected lay people understanding and special features of each professional group - Most of the pre-test participants were recruited through stakeholder channels - Throughout the write-up phase for the paper the advisory group significantly contributed to edits of the paper     All in all, the stakeholder group contributed in ensuring that the intervention, including its content and delivery, reflected the underlying theories as well as in communicating the research in a scientific article. |
| **4: Discussion**  Outcomes— Comment on the extent to which PPI influenced the study overall. Describe positive and negative effects | Stakeholder involvement in this study was very effective and influenced important aspects of the interventions.    This might have been related to several factors. Firstly, the stakeholder group consisted of key people in health promotion and motivational interaction. They were experts from different fields and had a versatile background in both research and practice. Many of them also had previous experience in being a part of an advisory group and participating in facilitated workshops. Also, the researchers had previous experience in conducting workshops and facilitating discussions in a meaningful way. Furthermore, the right processes were in place, as the stakeholders were involved from the beginning to the end of the intervention development allowing them to help shape the intervention and allowing them to contribute fully to the process. Finally, the stakeholders could commit to the collaborative nature of the development process and had time to allocate to participating in the workshop. All these aspects contributed to the positive impact that the involvement had on this study.    However, there were limitations. Despite the high attendance, not all stakeholders could make it to every workshop. In addition, the stakeholders did not receive compensation and for some volunteering might increase burden and lessen engagement. In terms of the feedback, they were asked to comment on materials on the spot and in front of the group, which might limit some of the participants' input. These things were taken into consideration by highlighting the value of the stakeholders participation and inviting comments also from those who had participated less in the conversations. |
| **5: Reflections**  Critical perspective— Comment critically on the study, reflecting on the things that went well and those that did not, so others can learn from this experience | All in all, the co-operation was fruitful and contributed to the final intervention. The biggest challenge in the process was covering a lot of content in short periods of time. The researchers had to be careful in choosing the key elements to present and discuss in order not to burden the stakeholders.    In similar future studies, numeric and written form feedback could be added as a feature. Numeric feedback could be used as a conversation starter and better capture how the stakeholders first react to the content. Written form feedback could aid those who do not want to discuss their views in public, |

**Reference**: Staniszewska, S., Brett, J., Simera, I., Seers, K., Mockford, C., Goodlad, S., Altman, D. G., Moher, D., Barber, R., Denegri, S., Entwistle, A., Littlejohns, P., Morris, C., Suleman, R., Thomas, V., & Tysall, C. (2017). GRIPP2 reporting checklists: Tools to improve reporting of patient and public involvement in research. *BMJ*, *358*, j3453. <https://doi.org/10.1136/bmj.j3453>

## Supplement 3. *Motivational interaction behaviours from the originally evaluated training mapped onto the newly optimised intervention (Task 1). (Note that MBCT name is the same as in the training if not spelled out)*

| **Motivational interaction behaviours in current training** | **Corresponding MBCTs (Teixeira et al., 2020)** | **Motivational technique (Renko et al., 2020; 2022)** | **Comments** |
| --- | --- | --- | --- |
| Open questions | (N/A) | Open questions & showing interest | Open questions included as a separate technique based on the positive feedback on the previous training even though not found independently in the MBCT taxonomy. Open questions are framed as a way to display interest and elicit discussion even though not mentioned in the technique name. |
| Provide a meaningful rationale | MBCT 5 | Providing structure  and rationale | Providing structure and rationale was the only technique in the initial training with a structuring element but it also included autonomy support. The technique was broken down into three separate techniques to ensure enough focus on the structuring techniques. |
| Clarify expectations | MBCT 16 |  |  |
| Help develop a clear and concrete plan of action | MBCT 19 |  |  |
| Reflective listening | MBCT 11. Demonstrate/show interest in the person & MBCT 12. Use empathic listening | Empathising and reflective listening | The first formulation was “Show interest and empathy” following the MBCT labels more closely. However, we concluded that reflective listening encompasses these aspects in a more concrete way. |
| Providing choice | MBCT 6 | Providing choice | No major changes |
| Use non-controlling, informational language | MBCT 3 | A style of understanding resistance and applying non-controlling language | No major changes |
| Offer constructive, clear and relevant feedback | MBCT 18 | Positive feedback & appreciation | Positive feedback & appreciation was divided into two separate techniques. “Positive feedback” was complemented with structuring elements.  “Appreciation” was complemented with an element of showing respect. This technique encompasses the respectful approach and unconditional regard in interaction as a whole. |
|  |  |  |  |
| Showing appreciation and respect | MBCT 8. Acknowledge and respect perspectives and feelings & MBCT 10. Show unconditional regard |  |  |
| N/A | N/A | Advising without pressing | The principle of advising without pressing was communicated along with other techniques: *non-controlling, informational language and in all structuring techniques* |

## Supplement 4. *Brief summary of findings from the literature review by Matosic et al.* (2016) *to inform the selection of behavioural determinants (Task 1).*

Multiple possible factors related to (de)motivational interaction behaviours have been identified in a review for those in positions of power/influence when interacting with subordinates in the areas of sports, education and family life (Matosic et al., 2016). The review identified factors under three main categories: contextual factors, perceptions of subordinates’ behaviours and motivation, and personal factors. The predominant contextual factors were the social-environmental factors and external pressures such as stress, social support or cultural norm. Perception of others’ behaviours related to e.g., the subordinates’ engagement level, moodiness and perceptions of motivation. Personal factors identified in the review included e.g., individuals’ beliefs about effectiveness, self-regulation, self-determined motivation, internal pressures (e.g., ego-involvement), and psychological need satisfaction.

## Supplement 5. *Participant view of the behaviour change process formulated as a bridge over the intention-behaviour gap (Task 2). The image was used throughout the intervention to visualise the change process and how the behaviour change activities relate to it.*

## Supplement 6. *Outline of the expected enactment of self-enactable techniques (Knittle et al., 2019) over the course of the MotiStyleSport intervention. (Note. Does not include behaviour change techniques delivered by the facilitator, or self-enactable techniques that were not explicitly instructed or prompted but participants might have used in their behaviour change attempts independently) (Task 2) S=Session, H=Homework, PS=Peer session*

| **Self-enactable technique (Knittle et al., 2019)** | **BCT enactment expectation in feasibility study** | **S1** | **H1** | **S2** | **H2** | **PS1** | **S3** | **H3** | **PS2** | **S4** | **H4** | **PS3** | **S5** | **H5** | **PS4** | **S6** | **H6** | **PS5** |
| --- | --- | --- | --- | --- | --- | --- | --- | --- | --- | --- | --- | --- | --- | --- | --- | --- | --- | --- |
| #1 Agenda mapping | Participants identify the motivational interaction behaviours that they want to focus on from a curated set of options. The motivational interaction behaviours can be either something new to adopt, or some de-motivational interaction behaviours the want to stop doing. | x | x | x | x |  | x | x |  | x | x |  | x | x |  | x | x |  |
| #5 Behavioural goal setting | Participants set behavioural goals for enacting the motivational interaction behaviours in their professional settings following the SMART principles.Participants start setting the goals during the session and finalise them after the session. |  |  |  |  |  |  |  |  | x | x |  |  |  |  | x | x |  |
| #7 Problem solving | Participants analyse barriers and facilitators for the motivational and demotivational interaction behaviours and plan strategies to overcome barriers and strengthen facilitators. They also form if-then plans and analyze case examples. |  |  | x |  |  | x |  |  | x |  |  | x |  | x | x |  |  |
| #8 Action planning | Participants make concrete and specific actions plans for self-monitoring and enacting the motivational interaction behaviours in their professional setting. Participants start making the plans during the session and finalize them after the session. | x | x | x | x |  | x | x |  | x | x |  | x | x |  | x |  | x |
| #9 Review behavioural goal(s) | Participants evaluate their plans and progress and adjust their goal(s) accordingly. |  |  |  | x |  |  | x |  |  | x |  |  | x |  |  | x | x |
| #11 Discrepancy between current behaviour and goal | Participants evaluate their current behaviour for each (de)motivational interaction behaviour, and compare it to their behavioural goals. |  |  |  |  |  |  |  |  | x |  |  |  | (x) |  |  |  |  |
| #13 Public commitment (extra option) | Participants are encouraged verbally and by tips in the workbook to talk about their motivational interaction behaviour change attempts in their professional settings, e.g., with their clients or colleagues. |  |  | (x) | (x) |  |  | (x) |  |  |  |  |  |  |  | (x) |  |  |
| #15 Obtain feedback on behaviour (extra option) | Participants are encouraged verbally and by tips in the workbook to ask for feedback on their motivational interaction behaviour from, e.g., their e.g. colleagues. |  |  | (x) | (x) |  |  | (x) |  |  |  |  |  |  |  | (x) |  |  |
| #17 Self-monitoring of behaviour | Participants follow the frequency of their (de)motivational interaction behaviours with the help of a self-monitoring sheet. |  | x |  | x |  |  | x |  |  | x |  |  | x |  |  | x |  |
| #18 Self-monitoring outcome(s) of behaviour | Participants follow what consequences their (de)motivational interaction behaviours have for themselves and others with the help of a self-monitoring sheet and evaluation templates. |  | x |  | x |  |  | x |  |  | x |  |  | x |  |  | x |  |
| #33 Behavioural experiments | Participants experiment with one motivational interaction behaviour at a time and execute the experiment between the sessions. |  |  |  | x |  |  | x |  |  | x |  |  | x |  |  | x |  |
| #34 Obtain information about antecedents | Participants map their own antecedents by drawing their own self-insight map of their motivational interaction behaviours. They consider antecedents that aid and hinder (de)motivational interaction behaviours. |  |  | x | x |  |  |  |  |  |  |  | x |  |  |  |  |  |
| #36 Obtain information about social consequences | Participants track how their motivational interaction behaviours affect their clients, athletes or students. |  | x |  | x |  |  | x |  |  |  |  |  |  |  |  |  |  |
| #39 Credible source | Participants learn motivational interaction behaviours from an expert facilitator as well as from videos of other motivational interaction behaviour and behavioural regulation experts. | x |  | x |  |  | x |  |  | x |  |  | x |  |  | x |  |  |
| #40 Make consequences more memorable | Participants write down the consequences of their (de)motivating behaviours on the workbook. |  | x |  | x |  |  | x |  |  |  |  |  |  |  |  |  |  |
| #41 Memory aids (extra option) | Participants are encouraged to review their detailed plans and the motivational interaction behaviour description before execution. |  | (x) |  | (x) |  |  | (x) |  |  |  |  |  |  |  |  |  |  |
| #46 Observe demonstration of the behaviour | The participants observe as the intervention provider shows examples of technique use both by acting out dialogue and by showing videos of example situations on the sessions. | x |  | x |  |  | x |  |  | x |  |  | x |  |  |  |  |  |
| #52 Support others | Participants form peer support groups where they exchange their experiences about practicing and using the techniques. |  |  |  |  | x |  |  | x |  |  | x |  |  | x |  |  | x |
| #54 Obtain practical social support (extra option) | Participants are encouraged verbally and by tips in the workbook to obtain practical support from different groups of people, e.g., peers, managers, friends, social media groups. Possibility to do this also in the peer group. |  |  |  | (x) |  |  |  |  |  |  |  |  |  |  |  | (x) |  |
| #55 Obtain emotional social support | Participants discuss e.g. experiences and feelings about practising and performing the motivational interaction behaviours with their peer group. |  |  |  |  | x |  |  | x |  |  | x |  |  | x |  |  | x |
| #56 Obtain instruction on how to perform the behaviour | Participants follow the facilitator performing and explaining the motivational interaction behaviours during the sessions. Participants also view videos of motivational interaction behaviour performance. | x |  | x |  |  | x |  |  | x |  |  | x |  |  | x |  |  |
| #57 Remind of outcome goal content | Participants think about their motivation to perform and maintain the motivational interaction behaviours by reminding themselves of desired outcomes. |  |  |  |  |  |  |  |  |  |  |  |  |  |  | x |  |  |
| #60 Prompts/cues | Participants identify relevant behavioural cues for creating if-then plans. (Participants have also the option to take the plans or templates as prompts/cues to action in to the interaction situation, which is prompted on the workbook.) |  | (x) |  | (x) |  | x | x |  | x | x |  | x | (x) |  |  |  |  |
| #67 Behaviour substitution | In the context of if-then planning, the participants can choose to substitute a demotivating interaction behaviour (or other unwanted reaction) with a motivating interaction behaviour of their choice (or something that facilitates motivational interaction behaviour, e.g., taking a reflection space). |  |  |  |  |  | x | x |  | x | x |  |  | x |  |  |  |  |
| #69 Behavioural practice or rehearsal | Participants practice using the motivating interaction behaviours with their peers by doing different exercises during the sessions and systematically repeat the behaviours in their professional context outside the sessions. | x |  | x | x |  | x | x |  | x | x |  | x | x |  | x | x |  |
| #70 Habit formation | Participants execute and systematically repeat their if-then plans in their professional setting whenever encountering the cues of their choice. |  |  |  |  |  |  | x |  |  | x |  |  | x |  |  | (x) |  |
| #71 Habit reversal | Participants execute and systematically repeat their if-then plan to substitute a demotivating interaction behaviour (or other unwanted reaction) with a motivating interaction behaviour of their choice (or something that facilitates the motivational interaction behaviour, e.g., taking a reflection space). |  |  |  |  |  |  | x |  |  | x |  |  | x |  |  |  |  |
| #74 Graded tasks | Participants set easy and clear tasks for practising motivational interaction behaviours and gradually build on what they have already learned and learn how to perform and combine the behaviours in more difficult situations. | x | x | x | x |  | x | x |  | x | x |  | x | x |  | x | x |  |
| #100 Reflect on reasons to perform the behaviour | Participants reflect on their personal reasons why performing the motivational interaction behaviours is important to them |  |  |  |  |  |  |  |  |  |  |  |  |  |  | x |  |  |
| #102 Find meaning in target behaviour | Participants reflect on how the motivational interaction behaviours link with their other values and self-image. |  |  |  |  |  |  |  |  |  |  |  |  |  |  | x |  |  |

## Supplement 7. *Behavioural determinants and how they were targeted in the MotiStyleSport intervention. (Task 2)*

| **Determinants for long-term use of motivational interaction target behaviours** | **How targeted in the MotiStyleSport intervention** | **Behaviour change techniques (BCTs; BCTTv1)** |
| --- | --- | --- |
| **Knowledge**: Knowledge and understanding of the principles of the motivational interaction behaviours and how to use them. | - Mini-lectures and video lectures by researchers on the theoretical background and the contents of motivational interaction behaviours - Videos and written examples of how to use motivational interaction behaviours - Small group / pair activities to try out the motivational interaction behaviours - Behavioural experiments in one’s own setting guided by homework templates and instructions | 4.1 Instruction on how to perform the behaviour  4.4 Behavioural experiments  6.1 Demonstration of the behaviour  8.1 Behavioural practice/rehearsal  9.1 Credible source |
| **Self-efficacy:** Belief that one can learn motivational interaction behaviours and confidence that one knows how to use motivational interaction in one’s own setting | - Activities that emphasise what one already knows and does well, and what one can build on. - Guidance on how to execute activities and homework. - Supportive feedback on activities and behavioural experiments by the intervention deliverer and others. - Sharing difficulties (and how to overcome them) to normalise them in group discussions and peer support groups. | 3.1. Social support (unspecified)  4.1. Instruction on how to perform the behaviour  6.1. Demonstration of the behaviour  8.1. Behavioral practice/rehearsal  15.3 Focus on past success |
| **Skills**  **to use motivational interaction behaviours:** Skills to use the motivational behaviours the desired way  **to self-regulate behaviour and to regulate emotions:** Skills to plan, experiment, self-monitor and self-evaluate one’s practice, and to regulate one’s emotions in interaction situations. | - Clear and detailed instructions how to use motivational interaction behaviours during sessions and across materials (also to take home) - Behavioural experiments and practice both during the sessions and in one’s own setting - Mini-lectures on the behaviour change process (depicted as a bridge over the intention-behaviour gap) and self-regulation skills (self-monitoring, planning and goal setting, execution, goal review, habit forming and breaking) - Homework utilising the self-regulation skills with the help of different templates for each skill - Mini-lecture and discussion on what emotion regulation is and how to do it - Individual task to make if-then plans for situations where emotion regulation is needed | 1.1. Goal setting (behaviour)  1.2. Problem solving  1.4. Action planning  1.5. Review behaviour goal(s)  2.3. Self-monitoring of behaviour  4.1. Instruction on how to perform the behaviour  4.4. Behavioral experiments  6.1. Demonstration of the behaviour  8.1. Behavioural practice/rehearsal 8.3. Habit formation  8.7. Graded tasks |
|  |  |  |
| **Outcome expectations:** Belief that the motivational interaction behaviour is beneficial and effective in interaction situations and PA/sports promotion. | - Mini-lecture on the general good outcomes of motivational interaction behaviours based on SDT and the circumplex model of de(motivational) styles - Self-monitoring the consequences of using motivational interaction behaviours in one’s own setting and sharing them in group discussions during sessions and peer support sessions | 2.4. Self-monitoring of outcome(s) of behaviour  5.3. Information about social and  environmental consequences |
| **Autonomous motivation:** Motivation to practice and use the motivational interaction behaviours because it is important for oneself personally | - Individual and pair/small group activities focusing on finding one’s own important reasons to practice and implement the behaviours into one’s practice - Homework templates include a section to write down reasons why it is important to one personally to do the homework | *No applicable BCTs in BCTTv1.* |
| **Self-insight of one’s motivational interaction behaviours:** Understanding of why one behaves the way one does in different situations | - Self-monitoring the situations that give rise to wanted and unwanted behaviours in one’s own setting - Drawing a self-insight map of one’s personal determinants for different motivational (and/or demotivational) interaction behaviours | 2.3. Self-monitoring of behaviour  4.2. Information about antecedents 7.1 Prompts/cues |
| **Norms:** Belief that other people (managers, colleagues, clients etc.) view the use of motivational interaction behaviours as acceptable and suitable | - Group discussions on the use and consequences of motivational and demotivational interaction behaviours in one’s own setting - Sharing good and bad experiences about the use of motivational interaction behaviours in group discussions and peer support groups - Normalising and being open about challenges during sessions and when giving feedback on activities/homework | 5.3. Information about social and environmental consequences 6.2 Social comparison  6.3. Information about others’ approval |

## Supplement 8. *Key quantitative figures by intervention element. Ratings of four or above indicate high acceptability, ratings below three indicate compromised acceptability (reversed scores for self-efficacy).*

|  | **Acceptability domain (Mean; median)** | | | | | | | | | | | | | | | |
| --- | --- | --- | --- | --- | --- | --- | --- | --- | --- | --- | --- | --- | --- | --- | --- | --- |
|  | **Affective attitude** | |  | **Burden** |  | **Intervention coherence** | | |  | **Opportunity costs** |  | **Perceived effectiveness** | | |  | **Self-efficacy** |
| **Intervention element** | I liked [element] | I would recommend [element] for my colleagues |  | [Element] required too much effort |  | [Element] was easy to understand and follow | Understood why [element] was covered | I was able to complete the [element] following the instructions |  | [Element] took too much time from other things |  | [Element] helped to understand [learning target] | I learned concrete ways to adopt interaction techniques in my daily work | I learned concrete interaction techniques to use in my daily work |  | I did not know how to do [element] |
| Antecedents mini lecture | 4.3; 4.0 |  |  |  |  | 4.2; 4.0 | 4.8; 5.0 |  |  |  |  | 4.5; 5 |  |  |  |  |
| Self-insight map v. 1 |  |  |  |  |  |  |  |  |  |  |  |  |  |  |  |  |
| Assignment | 2.8; 2.0 |  |  | 3.2; 3.0 |  | 2.6; 2.0 |  | 2.3; 2.5 |  |  |  | 2.9; 3.0 |  |  |  | 3.2; 3.0 |
| Recap discussion | 3.6; 4.0 |  |  |  |  |  |  |  |  |  |  | 3.3; 3.0 |  |  |  |  |
| Self-insight map v. 2 |  |  |  |  |  |  |  |  |  |  |  |  |  |  |  |  |
| Assignment | 4.1; 4.0 |  |  | 2.3; 2.0 |  | 3.3; 3.5 |  | 3.1; 3.0 |  | 1.4; 1.0 |  | 4.0; 4.0 |  |  |  | 1.8; 1.5 |
| Recap discussion | 3.9; 4.0 |  |  |  |  |  |  |  |  |  |  | 3.4; 3.0 |  |  |  |  |
| If-then planning |  |  |  |  |  |  |  |  |  |  |  |  |  |  |  |  |
| Mini-lecture | 4.3; 4.5 |  |  |  |  | 3.9; 4.0 | 4.0; 4.0 |  |  |  |  |  |  |  |  |  |
| If-then planning assignment | 3.8; 4.0 |  |  | 2.4; 3.0 |  | 3.8; 3.5 |  | 3.8; 3.5 |  |  |  | 3.5; 3.0 |  |  |  | 1.9; 1.5 |
| Recap discussion | 3.0; 3.0 |  |  |  |  |  |  |  |  |  |  | 2.9; 3.0 |  |  |  |  |
| Homework | 3.3; 3.0 |  |  | 3.0; 3.0 |  | 3.3; 3.0 |  |  |  |  |  | 3.5; 3.5 |  |  |  |  |
| Small group discussion | 4.1; 4.0 |  |  | 1.7; 2.0 |  | 4.2; 5.0 |  |  |  |  |  |  |  |  |  |  |
| Emotion regulation mini lecture | 4.8; 5.0 |  |  |  |  |  |  |  |  |  |  | 4.8; 5.0 |  |  |  |  |
| Training as a whole | 4.6; 5.0 | 4.5; 5.0 |  | 2.2; 2.0 |  | 4.0: 4.0 |  |  |  | 1.9; 2.0 |  | 4.6; 5.0 (of interaction techniques)  4.5;5.0 (of one's own interaction) | 4.6; 5.0 | 4.7; 5.0 |  |  |
| Behaviour change session assignments in general | 3.8; 4.0 |  |  |  |  | 3.6; 3.5 |  |  |  | 2.0; 2.0 |  |  |  |  |  |  |
| Homework in general | 3.8; 4.0 |  |  | 2.5; 2.5 |  |  |  | 3.3; 3.0 |  |  |  |  | 4.0; 4.0 |  |  |  |
| Homework templates |  |  |  |  |  | 3.3; 3.0 |  |  |  |  |  | 3.8; 4.0 |  |  |  |  |

## Supplement 9. *Challenges identified in the empirical testing based on facilitator observations, participant surveys and interviews, and optimisation recommendations.*

| **Area of challenge** | **Source(s)** | **Challenge identified / feedback**  **(Estimated severity: Mild* Moderate** Severe***)** | **Optimisation actions recommended and implemented by developers/facilitators for the feasibility study** |
| --- | --- | --- | --- |
| **SESSION 1** (Observations only) | | | |
| Technical issues | Facilitator observations | 1. Technical challenges take up unnecessary time * 2. Videos not playing properly ** 3. Instructions for assignments not accessible for all ** | 1. Take more preparation actions (e.g., breakout rooms ready beforehand) Organise facilitators practice sessions 2. Download videos beforehand to have an offline backup 3. Create a complete workbook instead of just homework templates Send materials beforehand and send only instructions via chat |
| Session structure: time management | Facilitator observations | 4. Introduction rounds take too much time* 5. Lecture parts take up too much time from interactive parts (SDT and circumplex content)** | 4. Have participants present themselves in small groups more in detail and share their expectations of the course, after which they present themselves to the whole group by only giving their name and profession; facilitator cuts in if needed  5. Shorten video lecture content (circumplex and SDT) and ask input and opinions from participants  Add an interactive task to try out a motivational interaction behaviour early on Prepare participants by saying in the beginning that this lecture will include much introductory material and videos, which is anyway important and more interactive parts will follow later |
| Content: videos | Participant interviews | 6. Video content showing examples of (de)motivational interaction behaviours too easy (**for one person, not for all)*** | 6. Prepare participants by saying that elements in video may be familiar to some but important to show basic issues to everybody in the intro lecture Highlight that the key is to showcase the differences between motivational and demotivational interaction behaviours |
| Participant engagement | Facilitator observations | 7. Participants not prepared for interaction (on the move, no video, no note taking equipment)*** | 7. Include information about participation requirements for interaction in the recruitment letter Send email reminders of requirements for interaction before and after session 1 |
| Homework | Facilitator observation | 8. Of the two homework option, the other motivational interaction behaviour (Giving a meaningful rationale) was too complicated this early in the training** | 8. Change the motivational interaction behaviour of providing a meaningful rationale into asking open questions. |
| **SESSION 2** | | | |
| Content: self-insight map | Questionnaire,  Participant interviews | 9. Self-insight map too difficult to do, participants get confused*** 10. Not enough time to do the map*** 11. Instructions too difficult and long** | 9. Provide a clear example and a clear template that includes instructions  10. Allocate more time to complete and discuss assignment  11. Facilitate the participants through a step-by-step example, and participants complete it a step at a time  Note: New version created and tested on session 5 |
| Session structure: general | Participant interviews, facilitator observations | 12. A lot of content covered fast; challenges in internalising** 13. Lectures/videos take up time from exercises and discussions** | 12. Move one of the autonomy supportive behaviours to a later session.  13. (Partly solved by item 12.) Remind that for some the videos contain a lot of new material and thus they are important to view |
| Session structure: general | Participant interviews, facilitator observations | 14. Transitions between topics not always clear* 15. Links between key content not always clear*** | 14. Use transition slides to improve clarity in all sessions (between target behaviours and topics)  15. Use different icons for different motivational interaction behaviours Use circumplex more often to indicate relationships between main target behaviours |
| **SESSION 3** | | | |
| Facilitation | Facilitator observations  Participant interviews | 16. Group discussions in a big (4-5 people) group (break-out rooms) take too much time in the online environment* | 16. Create smaller groups (2-3 people per group)  In general: manage time better (with break-out rooms and discussions by having exact time stamps for each activity and preparing the break-out rooms in advance |
| Session content: If-then | Participant interviews | 17. Language confusing (e.g., cue-behaviour association)** | 17. Simplify language and only talk about if-then plans Introduce the subject with habits in general and introduce if-then later rather than as a leading concept |
| Session structure: If-then assignment | Questionnaire | 18. Transition to a new section too fast*** 19. Not clear that assignment related to one’s own interaction behaviour change and detecting issues in one’s own interaction*** 20. Not enough time for assignment or discussion** | 18. Add clear transition to one’s own interaction behaviour change by presenting the behaviour change bridge and the step the content relates to  19. Create supporting material in the workbook for if-then planning (incl. examples, templates)  20. Pre-record lecture to manage time and make transition clear Shorten if-then planning lecture to include only three main cases and examples |
| **SESSION 4** |  |  |  |
| Session structure | Facilitator observations  Participant interviews | 21. Too much content for one session (had to postpone a complete section about change talk)*** | 21. Cover only change talk on this session Combine resistance & conflict sections and cover them together as one entity in session 5 (swapped with change talk) |
| **SESSION 5** |  |  |  |
| Session content | Facilitator observations | 22. Change talk does not fit into the flow of content if presented here* | 22. Present change talk section in session 4 (swapped with resistance and conflict section) Make sure to show how the techniques fit together with this entity |
| Session content: self-insight map | Participant interviews and observations | 23. Creating the map required a lot from participants as there were too many techniques and virtually endless situations to choose from*** | 23. Clarify template by removing the situations, but list all motivational interaction behaviours Instruct participants to choose one that they want to practice based on their previous assignments |
| **SESSION 6** |  |  |  |
| Receipt: Chaos & autonomy mix-up | Facilitator observation | 24. Participant started a discussion around “chaos can be good” that indicated misunderstanding in terms of autonomy support** | 24. Add more clarifications of autonomy support vs. chaos Remind that there can be rules in an autonomy supportive environment (whereas in chaos there are no or unclear rules) In case similar discussion arises: script a reminder that psychological safety is key: it defines autonomy support and is not present chaos |
| Session structure: self-motivation and social support | Facilitator observation | 25. Presented as a way forward when motivation wanes after course but could be integrated earlier* | 25. Introduce at the beginning of the workbook Include template in the workbook to write down one’s own self-motivation techniques Include template in the workbook for asking social support Remind the participants about these mid-intervention and at the end of the intervention |
| Session structure: Way forward | Facilitator observation | 26. Long term planning not covered in pre-testing due to lack of time*** | 26. Include long term planning section and a template in the workbook Shorten the section of theoretical overview of techniques |
| Session structure: Way forward | Interview | 27. A booster session was requested to go through the learning process and next steps** | 27. Add a component of participants planning “a booster session” with the peer group 1-2 months post intervention to discuss progress and experiences with the motivational interaction behaviours |
| **OVERALL/GENERAL** | |  |  |
| Engagement: Homework usually not done | Interviews, Facilitator observation | Reasons include:   28. Not enough time to do the homework between sessions*** 29. Templates liked but a barrier to take them into use (need to be printed by participants)*** 30. Difficult to orientate and hard to ask clarifying questions when materials are not seen beforehand*** 31. Templates seem to burdensome to fill in unless there is a lot of time (text-heavy)*** | 28. Reschedule to have 2 weeks between sessions  29. & 30. Create and send a workbook to participants to lower the barrier and have a chance to orientate beforehand  31. Remove text from the templates,  Make templates more simple, colorful and inviting  In addition:  Start filling homework templates during the sessions  Structure more peer support around the homework Provide meaningful rationale for doing the homework. |
| Peer support | Interviews | 32. Not included in the first parts of the course but tested later with good feedback*** 33. Unprompted requests in two interviews for more peer group meetings*** | 32. & 33. Start peer group meetings from session 2 onward Add peer group support to long-term planning also |
| Participant engagement during sessions | Facilitator/ observator observation | 34. Possible barrier to ask questions and provide comments during lecture parts on Zoom* | 34. Invite comments after every subject Ask comments by name after a group task Remind participants regularly to use the raise hand feature and/or the chat for comments and questions |
| Presentation of content | Interview | 35. Some slides crowded and too text-heavy** 36. Assignment instructions sometimes too fast and complicated** | 35. Remove text from slides / spread out to multiple slides Add animations so that slides do not suddenly have a lot to look at  36. Simplify and shorten the instructions Give step-by-step guidance in longer assignments |
| General comment; exercises | Participant surveys, open field | 37. More exercises and more time for trying out motivational interaction behaviours during the sessions** 38. More time for discussing the exercises during the sessions ** | 37. & 38. Exercises spread out more evenly around the training Practice facilitation for each session beforehand to ensure there is enough time for all exercises |
| General comment: engagement | Participant surveys, open field | 39. Everyone’s voice should be heard in the activity recaps* | 39. Not feasible because of time constraints, no optimisation actions. |
| General comment: exercises | Participant interviews | 40. Sometimes it is difficult to play a role, so more context and scripted interaction could be added to exercises * 41. On the other hand: sometimes the exercises do not feel fitting if they are overscripted * | 39. & 40. Provide both kinds of exercises and include examples (more scripted in the beginning, more freedom towards the end) Give freedom to choose case studies from curated options during session 6 |
| Technical issues | Participant interviews | 42. Technical issues are perceived frustrating (e.g., slow transitions, difficulties with sharing materials through chat)** 43. Group assignment materials sent through chat do not open if not on Zoom app** | 42. Have a plan b for most common technical issues  Organise facilitators practice sessions  43. Avoid sending materials through chat, only instructions Instructions to take pictures of slides before going to the break-out room if needed Create and send beforehand a workbook with discussion and case templates also for break-out rooms |
